# Infection prevention and control in nursing severe coronavirus disease (COVID-19) patients during the pandemic

**DOI:** 10.1186/s13054-020-03076-1

**Published:** 2020-06-12

**Authors:** Lei Ye, Shulan Yang, Caixia Liu

**Affiliations:** 1 Department of Critical Care Medicine, Zhejiang Hospital, 12 Lingyin Road, Hangzhou, 310013 China; 2 Department of Nursing, Zhejiang Hospital, 12 Lingyin Road, Hangzhou, 310013 China

**Keywords:** COVID-19, Infection prevention and control, Critically ill patients, Pandemic

The novel coronavirus disease 2019 (COVID-19) is now worldwide publicity. Five to 20% of the total COVID-19 positive cases required admission to an intensive care unit (ICU) and the mortality rate was approximately 50% among critically ill patients who developed acute respiratory distress syndrome [1–5]. Deeply concerned by the spread and severity, the World Health Organization (WHO) characterized COVID-19 as a pandemic in March 2020. In February, Wuhan was facing a sudden shortage of health workers induced by the COVID-19 pandemic. The Chinese health authorities reported that 3019 Chinese health workers were infected with COVID-19, of which 10 died [6]. Front-line health workers are at high risk of infection. Inadequate awareness and precautionary measures, patient overload, and staff burnout are considered as relevant reasons for health worker infections. As an emergency measure, the China government dispatched 189 national medical teams comprising more than twenty-thousand health workers from all over the country who volunteered to combat COVID-19 in Hubei. They had been working together with local health workers and successfully controlled the development of the epidemic. The goal of “Zero” COVID-19 infection among health workers was achieved. Actually, from February 12 to April 9, 9282 health worker COVID-19 cases were reported by the US Center for Disease Control and Prevention, and contacts with COVID-19 patients in health care, household, and community settings were all detected [7]. COVID-19 infections among health workers are common and fatal to the health system. Infection among health workers may cause widespread transmission within the system and even lead to the collapse of the whole services. And this was what exactly happened in Harbin in the past weeks; a persisting cluster centered on an 87-year-old inpatient infected more than eighty people, including 8 health workers. The affected hospital urgently suspended routine medical services as a result.

Based on Wuhan’s experience, it is critical to develop tailored infection prevention and control (IPC) protocols for both workplace and non-occupational settings and to conduct effective IPC training. Thus, the following suggestions were summarized based on the first-hand experience of a national medical team from Zhejiang, to facilitate the development of IPC protocols in critical care settings.

## Appropriate implementing of PPE

**Fig. 1:**
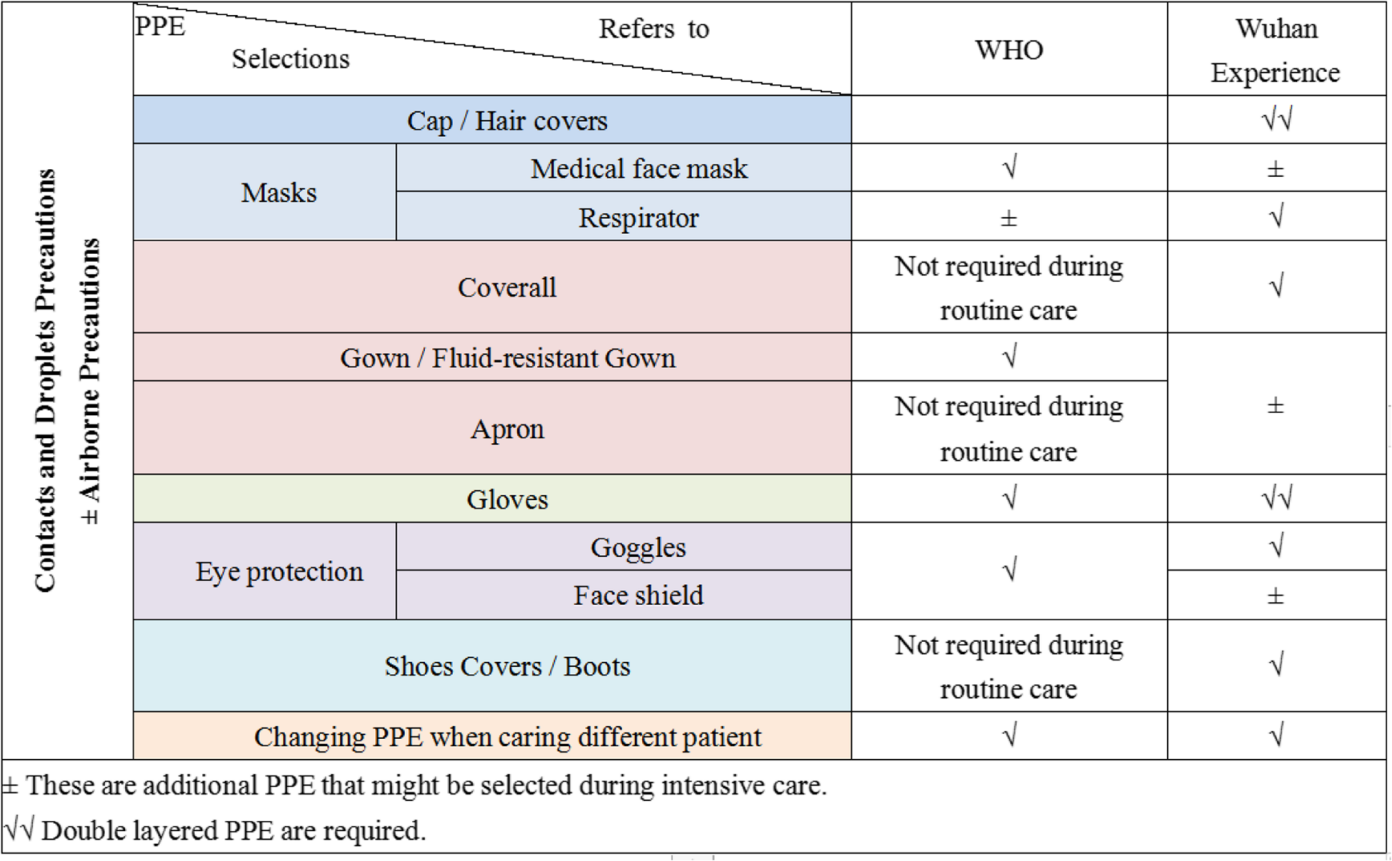
PPE selections for ICU setting

Generally, all health workers should implement appropriate personal protective equipment (PPE) regarding contact and droplet precautions based on recommendations by WHO [8]. For health workers in ICU, advanced protections are required during routine intensive care and airborne precautions are considered as airborne transmission may happen during aerosol-generating procedures. The implementation of PPE may be different by option in certain practices. In our experience, the most protective choices were made and the “zero” medical infection rate was treated as the top priority that all staff were equipped from head to toe. Compared to official recommendations, we selected some additional PPE during intensive care, such as an extra medical face mask outside the respirator, and both face shield and goggles (see Fig. 1). Additional PPE may increase the risk of sharp injuries and increase the difficulty of donning and doffing. To lower the incidence of adverse events, sequences of donning and doffing PPE were carefully developed based on the above selections through thorough group discussions and agreement was reached among the team.

## Donning and doffing PPE under the three-zone double-channel structure

**Fig. 2:**
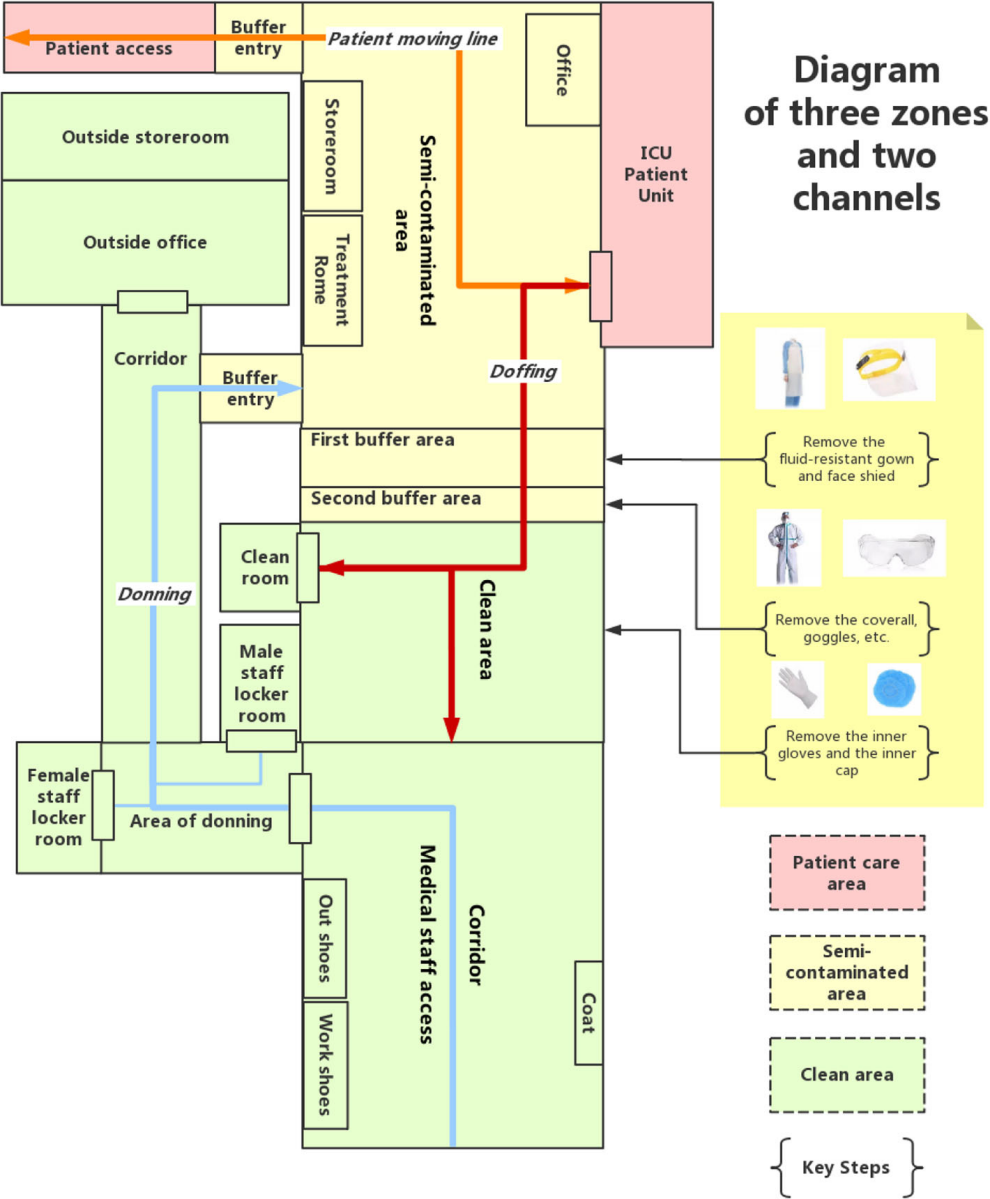
Diagram of the three-zone two-channel structure

The ward was reconfigured into a three-zone double-channel structure before accepting COVID-19 patients. In this design, the ward was divided into several working areas according to cleanliness and the moving lines of patients and medical staff were fixed (see Fig. 2). The patient care area was identified as contaminated, and all staff were fully equipped with PPE before entering the buffer area. When doing doffing, all staff took off the additional PPE during intensive care (such as the fluid-resistant gown and face shield) in the first buffer area that was near the patient care area. In the second buffer area, staff doffed the coverall and goggles. Finally, in the clean area, all staff removed the remaining PPE and conducted personal hygiene. We also developed reasonable shift rotations determined by the most tolerable shift lengths to prolong the use of PPE. In a 4–6 h shift, health care workers avoided eating, watering, and toileting.

## Real-time on-site IPC surveillance

To strengthen IPC, an inspector was set to facilitate the routine IPC management by on-site monitoring. Basically, the inspector was responsible for supervising the adherence of donning and doffing procedures of each health worker and real-time surveillance. In this way, some high-risk intensive interventions were identified and improvement measures were implemented promptly. COVID-19-specific precautions were drawn among the team consequently, such as waste management. According to recent reports, not only respiratory specimens but also serum, urine, and stool specimens might be positive for COVID-19. Even though no further IPC advice was provided, advanced procedures for waste managing were necessary, such as collecting respiratory and non-respiratory wastes in covered containers filled with chlorinated disinfectants and discarding in fastened double-layered medical waste garbage bags.

## IPC management in non-occupational settings

Timely after arriving Wuhan, we established the ICP team and developed our own practical ICP procedures in non-occupational settings as well. We strictly ruled our behaviors during traffic routes and in the residential region and facilitated the whole team with a remote communication and collaboration platform using cellphone applications to strengthen communication. Same as what we do in the ward, we established the three-area double-channel structure and fixed our moving line. Besides, we developed behavior codes among the team, such as limiting gatherings and personnel contacts, routine disinfection of contact surfaces (handphone, doorknob, handle, etc.), and frequent hand hygiene on certain occasions.

## Conclusions

All information provided in this paper is to strengthen the clinical practice in critical care settings and to better protect front-line health workers in nursing severe COVID-19 patients. The “zero” medical infection rate in our experience was hard won but worth fighting for.

## Data Availability

Data sharing not applicable to this article as no datasets were generated or analyzed during the current study.
